# A hierarchical spike-and-slab model for pan-cancer survival using pan-omic data

**DOI:** 10.1186/s12859-022-04770-3

**Published:** 2022-06-17

**Authors:** Sarah Samorodnitsky, Katherine A. Hoadley, Eric F. Lock

**Affiliations:** 1 Division of Biostatistics, University of Minnesota, Minneapolis, USA; 2 Department of Genetics, Lineberger Comprehensive Cancer Center, University of North Carolina at Chapel Hill, Chapel Hill, USA

**Keywords:** Bayesian hierarchical modeling, Bidimensionally-linked matrices, Pan-omics, pan-cancer, Spike-and-slab priors, survival analysis, The Cancer Genome Atlas (TCGA )

## Abstract

**Background:**

Pan-omics, pan-cancer analysis has advanced our understanding of the molecular heterogeneity of cancer. However, such analyses have been limited in their ability to use information from multiple sources of data (e.g., omics platforms) and multiple sample sets (e.g., cancer types) to predict clinical outcomes. We address the issue of prediction across multiple high-dimensional sources of data and sample sets by using molecular patterns identified by BIDIFAC+, a method for integrative dimension reduction of bidimensionally-linked matrices, in a Bayesian hierarchical model. Our model performs variable selection through spike-and-slab priors that borrow information across clustered data. We use this model to predict overall patient survival from the Cancer Genome Atlas with data from 29 cancer types and 4 omics sources and use simulations to characterize the performance of the hierarchical spike-and-slab prior.

**Results:**

We found that molecular patterns shared across all or most cancers were largely not predictive of survival. However, our model selected patterns unique to subsets of cancers that differentiate clinical tumor subtypes with markedly different survival outcomes. Some of these subtypes were previously established, such as subtypes of uterine corpus endometrial carcinoma, while others may be novel, such as subtypes within a set of kidney carcinomas. Through simulations, we found that the hierarchical spike-and-slab prior performs best in terms of variable selection accuracy and predictive power when borrowing information is advantageous, but also offers competitive performance when it is not.

**Conclusions:**

We address the issue of prediction across multiple sources of data by using results from BIDIFAC+ in a Bayesian hierarchical model for overall patient survival. By incorporating spike-and-slab priors that borrow information across cancers, we identified molecular patterns that distinguish clinical tumor subtypes within a single cancer and within a group of cancers. We also corroborate the flexibility and performance of using spike-and-slab priors as a Bayesian variable selection approach.

## Background

### Motivating application

Since its completion in 2018, the Cancer Genome Atlas (TCGA) database has become a cornerstone for studying the relationship between cancer molecular heterogeneity and clinical outcomes. TCGA contains data from multiple “omics” sources, including the genome, transcriptome, proteome, and epigenome, from over 10,000 patients across 33 types of cancer [1], opening the door to pan-omics, pan-cancer research. Changes to genomic function that affect cancer development and behavior occur at multiple omics levels, motivating several pan-omics studies that have discovered vast molecular variation across multiple levels within a single cancer type [2–4]. Meanwhile, pan-cancer research has been motivated by discoveries of the same genomic changes affecting tumors from different tissues-of-origin [5]. These discoveries suggest the importance of considering multiple omics sources and multiple cancer types at once to holistically characterize cancer’s etiological landscape.

One approach to studying molecular heterogeneity across both omics sources and cancer types is BIDIFAC+, a method of simultaneous factorization and decomposition of variation across bidimensionally linked matrices [6]. BIDIFAC+ identifies latent factors, analogous to principal components, that may be shared across any number of omics platforms or sample sets. These components describe patterns of variability across these combinations of omics sources and patient groups. When applied to TCGA data, BIDIFAC+ revealed patterns of variability shared by mRNA, miRNA, methylation, and protein data driving heterogeneity across multiple cancers [6]. However, these results were solely exploratory, and did not consider prediction of important clinical endpoints. Our goal is to assess the prognostic value and clinical relevance of pan-omic patterns of molecular variability identified by BIDIFAC+. To do so, we sought to use a comprehensive model for overall survival that flexibly borrows information across the different types of cancer.

### Components of our pan-cancer, pan-omics analysis

Our approach builds on two active areas of statistical methodology: prediction via integrative dimension reduction (described in the “Prediction via bidimensional dimension reduction” section ) and structured Bayesian variable selection (described in the “Bayesian hierarchical spike-and-slab survival model” section).

#### Prediction via bidimensional dimension reduction

Predictive modeling in the case of a single high-dimensional dataset often begins by first applying a method such as principal components analysis (PCA) to obtain a small set of latent variables (i.e., components) that explain variation in the data [7]. These components can be used for predictive modeling using classical approaches [8]. However, PCA does not translate smoothly to the multi-source (e.g., multi-omics) context. In this context, one may use the results of multi-source integrative methods, like joint and individual variation explained (JIVE) [9], structural learning and integrative decomposition (SLIDE) [10], or generalized integrative principal components analysis (GIPCA) [11]. These methods identify components that are shared across or specific to multiple sources, which has been shown to improve power and interpretation for multi-omics predictive models over ad-hoc applications of PCA [12]. However, these approaches do not apply when there are multiple sources of covariates and multiple sample sets, as is the case in the pan-omics, pan-cancer setting. This article addresses the issue of prediction across multiple sources of data and multiple sample sets by using components identified by BIDIFAC+ in a predictive model. BIDIFAC+ identifies components that may be shared across any number of sources (e.g., omics platforms) and any number of sample sets (e.g., cancer types). In particular, we use BIDIFAC+ components from bidimensional integration of multiple omics sources and multiple cancer types to model TCGA patients’ overall survival (OS).

#### Bayesian hierarchical spike-and-slab survival model

In order to model the relationship between patient OS and components from BIDIFAC+ dimension reduction, we consider a Bayesian hierarchical survival regression framework. Bayesian hierarchical regression has been used previously for pan-cancer survival modeling [13], and is attractive in this context because it facilitates borrowing of information across cancer types while allowing a different survival model for each cancer. This feature of our approach is motivated by the assumption that molecular patterns may drive heterogeneity in more than one cancer. However, our model is also flexible enough to allow the effect of these patterns to differ according to the cancer type. Accommodating a censored outcome is straightforward in this framework, which has been demonstrated in prior work [13, 14].

Many genomic components have little relation to clinical outcomes, and so we pursued a sparse model that accommodates variable selection within the hierarchical framework. There is an extensive literature on Bayesian approaches to variable selection. Mitchell and Beauchamp [15], George and McCulloch [16], and Kuo and Mallick [17] are foundational but differing perspectives on spike-and-slab variable selection. The spike-and-slab approach is unique in providing an “included/excluded” interpretation for each predictor through the use of indicator variables that turn on and off each coefficient. In contrast, other Bayesian variable selection approaches adaptively shrink coefficients of uninformative predictors towards zero. Examples of such priors include the Bayesian lasso [18], the Bayesian elastic net [19], and the horseshoe prior [20].

These Bayesian variable selection methods have been used in hierarchical models of many forms. Yang et al. [21] propose using spike-and-slab priors to identify important groups of covariates in nonparametric regression models and seemingly unrelated regressions models. Zhang et al. [22] propose a variable selection approach which identifies groups of covariates to include in the model and estimates lasso solutions for coefficients in selected groups. These methods operate on a single sample set where inducing sparsity at the group level on covariates is desired. In contrast, Suo et al. [23] and Mousavi et al. [24] demonstrate using spike-and-slab priors for variable selection on a single covariate set shared by multiple sample sets for classification. Hierarchical variable selection has also been considered in Bayesian survival models, as is done in Lee et al. [25] and Lee et al. [26], which both present the use of spike-and-slab priors in proportional hazards models. Maity et al. [14] consider the pan-cancer context and induce sparsity in a Bayesian model for survival using hierarchical horseshoe priors.

To induce sparsity in our context across multiple cancer types (i.e., groups) we extend George and McCulloch [16]’s definition of a spike-and-slab prior in three ways: (a) we allow the possibility that a predictor is included for one sample group but not another, (b) we allow the slab distribution’s location and scale to be inferred hierarchically based on data from groups for which the covariate is included, and (c) we impose a prior on the inclusion probabilities of each covariate to borrow information across groups. These modifications adapt the original formulation to borrow information across groups without compromising the flexibility that covariate inclusion and coefficient estimation can differ between groups. This approach is attractive when groups offer agreeable information about shared covariates, and it is advantageous to borrow information to increase power. Our model accommodates a potentially-censored outcome, offering a translational approach that isolates predictors informative of survival.

Our context differs from Yang et al. (2020) [21] and Zhang et al. (2014) [22], who study variable selection on a shared covariate set for a single sample set. Our approach also differs from Mousavi et al. (2014) [24] and Suo et al. (2013) [23], who study variable selection for multiple sample sets but require the same predictors be included for all sample sets. The approach of Maity et al. (2020) [14] most closely resembles ours by borrowing strength across multiple sample sets to select informative predictors for patient survival but allowing for differences in the model for each sample set. However, we consider a spike-and-slab prior as opposed to a horseshoe prior to facilitate an “inclusion/exclusion” interpretation for each predictor with accompanying posterior probabilities, which is useful in this context. Our work also differs in that we allow for the survival model for each cancer type to depend on a different set of predictors.

The rest of our article is organized as follows. In the “Methods” section we describe our methods in detail, including an introduction to the BIDIFAC+ method, the spike-and-slab prior, and our hierarchical extensions. In the “Application to pan-cancer, pan-omics data” section, we apply our Bayesian model to TCGA data to predict patient OS using patterns of variability identified by BIDIFAC+ and investigate the clinical relevance of the results. In the “Simulation study” section, we present a simulation study evaluating the flexibility of the hierarchical spike-and-slab prior in the context of our data application. We provide a discussion of the results and suggestions for future work in the “Discussion” section and concluding thoughts in the “Conclusion” section.

## Methods

Here we describe our Bayesian hierarchical model with spike-and-slab priors. We first provide an overview of bidimensionally-linked data and the BIDIFAC+ method in the “BIDIFAC+ for bidimensionally-linked data” section. We then describe the classical spike and slab model in the “Spike-and-slab priors” section. In the “Hierarchical extensions” section, we describe how to extend the spike-and-slab prior to borrow information across grouped data. Then, we outline our full model used for the survival outcome in “Extensions to survival data” section. We describe computation of the posterior predictive log-likelihood for model assessment in the “Posterior predictive likelihood validation” section.

### BIDIFAC+ for bidimensionally-linked data

**Fig. 1:**
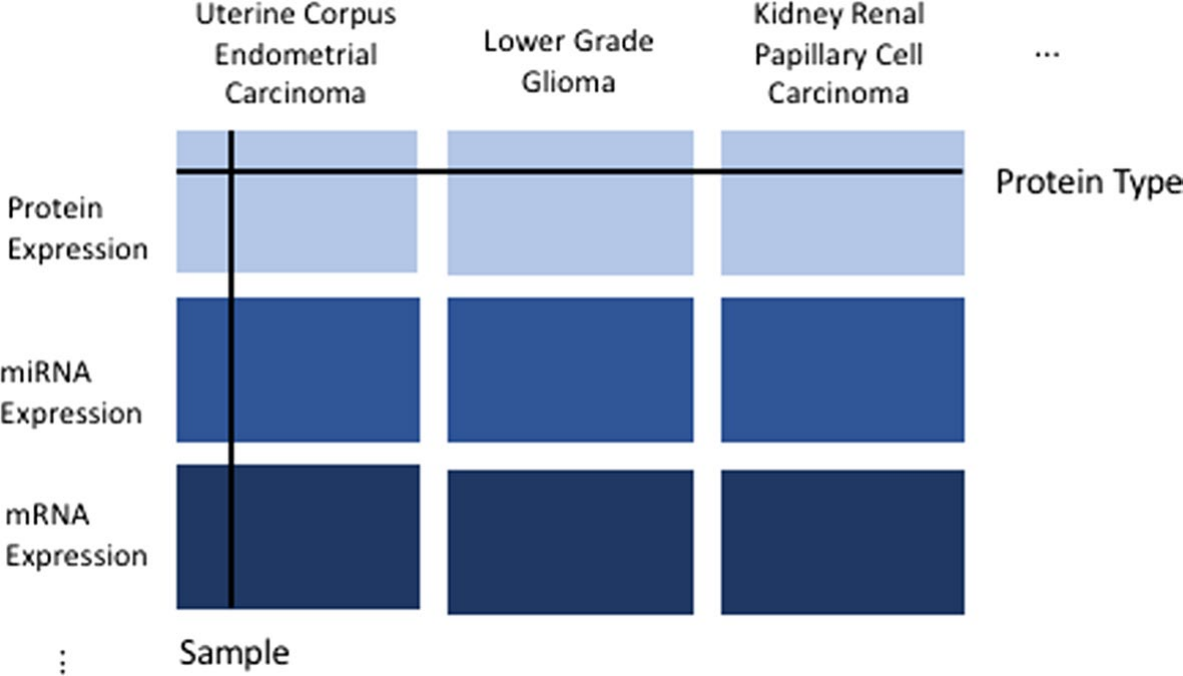
Visualization of bidimensionally-linked data. Each rectangle represents a set of omics features for a given cancer type. Each row corresponds to an omics feature (e.g., protein, gene, etc.) and each column to a sample

Here we briefly introduce the BIDIFAC+ method. BIDIFAC+ [6] is an exploratory factorization method for *bidimensionally-linked data*. Bidimensionally-linked data is a data structure consisting of several datasets linked by their rows (e.g., shared omics features) and their columns (e.g., shared patient cohorts), as visualized in Fig. 1. BIDIFAC+ decomposes bidimensionally-linked data into a series of low-rank modules or matrices explaining structured variability shared either globally (across all datasets), across the row sets, across the column sets, or unique to each dataset. To illustrate the BIDIFAC+ decomposition, define the following concatenated matrix:

1 $$\begin{aligned} \mathbf {X}_{\cdot \cdot } = \begin{pmatrix} \mathbf {X}_{11}&{} \mathbf {X}_{12}&{} \dots &{}\mathbf {X}_{1I} \\ \vdots &{} \vdots &{} \ddots &{} \vdots \\ \mathbf {X}_{J1} &{} \mathbf {X}_{J2} &{} \dots &{} \mathbf {X}_{JI} \end{pmatrix} \end{aligned}$$

where each $\mathbf {X}_{ji}$ represents a dataset from omics platform $j,j=1,\dots , J$ and patient cohort $i, i=1,\dots , I$. The BIDIFAC+ factorization is:

2 $$\begin{aligned} \mathbf {X}_{\cdot \cdot } = \sum _{k=1}^K \mathbf {S}_{\cdot \cdot }^{(k)} + \mathbf {E}_{\cdot \cdot } \; \text {where} \; \mathbf {S}_{\cdot \cdot }^{(k)} = \begin{pmatrix} \mathbf {S}_{11}^{(k)}&{} \mathbf {S}_{12}^{(k)}&{} \dots &{}\mathbf {S}_{1I}^{(k)} \\ \vdots &{} \vdots &{} \ddots &{} \vdots \\ \mathbf {S}_{J1}^{(k)} &{} \mathbf {S}_{J2}^{(k)} &{} \dots &{} \mathbf {S}_{JI}^{(k)} \end{pmatrix} \end{aligned}$$

$\mathbf {S}_{\cdot \cdot }^{(k)}$ is a low-rank module which corresponds to structured variation that exists on a subset of the *J* omics sources and *I* patient groups and $\mathbf {E}_{\cdot \cdot }$ represents random noise. The submatrix $\mathbf {S}_{ji}^{(k)}$ of $\mathbf {S}_{\cdot \cdot }^{(k)}$ is non-zero if the *k*th low-rank module explains variability in omics source *j* and cancer *i*, or entirely 0 otherwise. The number of low-rank modules, *K*, is either set to $K = (2^I-1)(2^J-1)$ to enumerate all combinations of omics platforms and cohorts, or may be chosen such that the modules explain a pre-determined amount of variability in the data.

To derive predictors from these low-rank modules, we obtain the sample scores via the SVD of each $\mathbf {S}_{\cdot \cdot }^{(k)}$. These sample scores reflect how the identified multi-omic patterns are expressed in samples across patient cohorts. To understand the biological relevance of these multi-omic patterns, we may investigate the loadings from this SVD, which map the latent patterns to the observed feature space. Investigating the loadings reveals the observed molecular features that are most relevant to the identified multi-omic pattern.

### Spike-and-slab priors

We now introduce the spike-and-slab prior in its general form. Consider the ordinary linear model for an outcome $y_i$ given covariates $\{X_{i\ell }\}_{\ell =1}^L$,

3 $$\begin{aligned} y_{i}=\beta _0+\sum _{\ell =1}^L \beta _{\ell } X_{i\ell }+\epsilon _{i} \end{aligned}$$

for subjects $i=1,\ldots ,I$. The classical spike-and-slab model considered by George and McCulloch [16] imposes the following prior on the coefficients $\beta _\ell$:

4 $$\begin{aligned} \begin{aligned} \beta _\ell | \gamma _\ell&\sim (1-\gamma _\ell ) \hbox {N}(0, \tau _\ell ^2) + \gamma _\ell \hbox {N}(0, c_\ell ^2 \tau _\ell ^2) \\ \gamma _\ell | \pi _\ell&\sim \hbox {Bernoulli}(\pi _\ell ) \end{aligned} \end{aligned}$$

where $\tau _\ell ^2$ is chosen to be small and $c_\ell ^2$ is chosen to be large. The indicator $\gamma _\ell$ reflects from which distribution $\beta _\ell$ is generated: if $\gamma _\ell =1$, $\beta _\ell$ is generated from the slab, $\hbox {N}(0, c_\ell ^2 \tau _\ell ^2)$, and if $\gamma _\ell =0$, $\beta _\ell$ is generated from the spike, $\hbox {N}(0, \tau _\ell ^2)$. Practically, $\gamma _\ell$ indicates whether covariate $\ell$ has a non-negligible contribution to the predictive model. The prior encourages sparsity via the spike, and shrinks coefficients under the slab towards zero. Uncertainty in model selection is easy to interpret via the posterior probabilities of each $\gamma _\ell$.

### Hierarchical extensions

Now, assume the data are grouped or clustered, e.g., by genetic strain or cancer type. We index each group by *i*, $i=1,\dots , I$ and index subjects within each group by *j*, $j=1,\dots , n_i$ where $n_i$ is the sample size for group *i*. Consider *L* covariates $\{X_{1}, X_{2}, \dots , X_{L}\}$, where a subset of the *L* covariates is available for each group. Let $S_i = \{\ell : X_\ell \text { exists for group } i \}$ be the indices for covariates measured on group *i*. Let $y_{ij}$ be the response for the *j*th subject in the *i*th group, $j = 1, \dots , n_i$, $i = 1, \dots , I$. Specify a linear model for $y_{ij}$ as follows:

5 $$\begin{aligned} y_{ij} = \beta _{i0} + \sum _{\ell \in S_i} \beta _{i\ell } X_{ij\ell } + \epsilon _{ij} \end{aligned}$$

where $\epsilon _{ij}$ are iid random variables such that $\mathbb {E}(\epsilon _{ij}) = 0$ and $\hbox {Var}(\epsilon _{ij}) = \sigma ^2$. This framework not only allows for covariate sets to differ between groups, but also allows the effect of each predictor to vary by group, where the partial effect of predictor $\ell$ for group *i* is given by $\beta _{i\ell }$. We allow for the possibility that a predictor may have no effect on group *i*’s outcome through the use of spike-and-slab variable selection. We extend George and McCulloch’s implementation of a spike-and-slab prior (4) by inferring the distribution of the slab hierarchically (with a possibly non-zero mean) while allowing for differential inclusion across groups. The hierarchical structure is also extended to the inclusion probabilities. We define our spike-and-slab prior as follows:

6 $$\begin{aligned} \begin{aligned} \beta _{i\ell }|\tilde{\beta }_\ell , \lambda ^2_\ell , \gamma _{i\ell }&\sim (1-\gamma _{i\ell }) \hbox {Normal}\left( 0, z^2\right) + \gamma _{i\ell } \hbox {Normal}(\tilde{\beta }_\ell , \lambda ^2_\ell ) \\ \tilde{\beta }_\ell&\sim \hbox {Normal}(0, \tau ^2) \\ \lambda ^2_\ell&\sim \hbox {Inverse-Gamma}(\alpha _1, \alpha _2) \\ \gamma _{i\ell }|\pi _\ell&\sim \hbox {Bernoulli}(\pi _\ell ) \\ \pi _\ell&\sim \hbox {Beta}(1,1) \end{aligned} \end{aligned}$$

where $\ell = 1,\dots , L$ and $z^2$ is chosen to be very small. Here, $\gamma _{i\ell }$ is an inclusion indicator that reflects whether or not the coefficient for covariate *ℓ* comes from the spike or the slab distribution for group *i*. If $\gamma _{i\ell } = 1$, then $\beta _{i\ell }$ is generated from the slab, $\hbox {Normal}(\tilde{\beta }_\ell , \lambda ^2_\ell )$, and if $\gamma _{i\ell } = 0$ then $\beta _{i\ell }$ is generated from the spike, $\hbox {Normal}\left( 0, z^2\right)$. Data from clusters for which covariate $\ell$ is generated from the slab are used to infer the mean $\tilde{\beta }_\ell$ and variance $\lambda ^2_\ell$ of the slab distribution. This may increase our power to infer covariate $\ell$’s effect if the groups provide concordant information. We apply a Beta prior to the inclusion probability $\pi _\ell$ for covariate $\ell$, cementing a fully Bayesian framework. A unique inclusion probability for each predictor induces correlation between selected predictors across the *I* groups. Consequently, inference on $\pi _\ell$ reflects the proportion of groups for which covariate $\ell$ has predictive power.

### Extensions to survival data

We now outline our full hierarchical spike-and-slab survival model with the likelihood and hyperparameters we use in our data application and simulations. Let $y_{ij}$ be the survival time, which may or may not be observed due to censoring, for the *j*th subject in the *i*th group, $j = 1, \dots , n_i$, $i = 1, \dots , I$. Let $S_i = \{ \ell : X_\ell \text { exists for group } i \}$ be the set of covariate indices available for group *i*. It is possible that group *i* and group $i'$ where $i\ne i'$ do not share all of the same covariates. Further, define

7 $$\begin{aligned} y^{*}_{ij} = {\left\{ \begin{array}{ll} y_{ij} \quad \text {if subject is not censored} \\ y_{ij}^{c} \quad \text {if subject is censored}\end{array}\right. } \end{aligned}$$

where $y_{ij}^c$ is the censor time for the *j*th subject in the *i*th cancer type. Then, our hierarchical spike-and-slab model is

8 $$\begin{aligned} \begin{aligned} \log y_{ij}&\sim \text{ Normal } \left( \beta _{0i} + \sum _{\ell \in S_i} \beta _{i\ell } X_{ij\ell }, \sigma ^2 \right) \\ \beta _{0i}|\tilde{\beta }_0, \lambda ^2_0&\sim \hbox {Normal}(\tilde{\beta }_0, \lambda ^2_0)\\ \beta _{i \ell }|\gamma _{i\ell }, \tilde{\beta }_\ell , \lambda ^2_\ell&\sim (1-\gamma _{i\ell }) \hbox {Normal}\left( 0, \frac{1}{10000}\right) + \gamma _{i\ell }\hbox {Normal}(\tilde{\beta }_\ell , \lambda ^2_\ell ) \\ \gamma _{i\ell } | \pi _\ell&\sim \hbox {Bernoulli}(\pi _\ell ), \pi _\ell \sim \hbox {Beta}(1,1) \\ \tilde{\beta }_0&\sim \hbox {Normal}(0, 10^2), \lambda ^2_0 \sim \hbox {Inverse-Gamma}(1, 1) \\ \tilde{\beta }_\ell&\sim \hbox {Normal}(0,1), \lambda ^2_\ell \sim \hbox {Inverse-Gamma}(5,1) \\ \sigma ^2&\sim \hbox {Inverse-Gamma}(0.01, 0.01) \end{aligned} \end{aligned}$$

where $$\ell \in S_i$$ for the *i*th cancer type and *j* indexes the subject within the *i*th cancer type. We selected a log-normal likelihood because previous work demonstrated it outperforms other parametric models for TCGA pan-cancer survival [13, 14]. The priors for $$\tilde{\beta }_0$$, $$\lambda ^2_0$$, $$\tilde{\beta }_\ell$$, and $$\lambda ^2_\ell$$ were chosen to be sufficiently uninformative and to match the scale of the data, though our later data application results in the “Application to pan-cancer, pan-omics data” section appeared to be insensitive to the choice of hyperparameters in these priors. The spike variance was arbitrarily set at $$\frac{1}{10000}$$ and results were not sensitive to this choice. We implement our model using an in-house Gibbs sampling algorithm, in which the unobserved outcomes $$y_{ij}$$ are simulated from their full conditional distribution when the observation is censored ($$y_{ij}^*=y_{ij}^c)$$. All full conditional distributions for the censored survival model used for our data application in the “Application to pan-cancer, pan-omics data” section are provided in Additional file 1: Appendix A .

### Posterior predictive likelihood validation

We assess our hierarchical spike-and-slab approach and related models via 5-fold cross validation of the log-posterior predictive likelihood. The log-posterior predictive likelihood is a measure of the predictive accuracy of the model. We use this measure in a cross validation framework as a holistic way to assess the predictive ability of a model fit to training data on an independent test set. The log-posterior predictive likelihood is defined as follows. For $k=1,\dots , 5$, consider the *k*th training-test set split, $\vec {Y} = \{\vec {Y}^{\text {train}}_k, \vec {Y}^{\text {test}}_k \}$. Let $p(y|\Theta _0, X)$ be the log-normal probability density for survival time, given all model parameters $\Theta _0$ and covariates *X*. Define $X^{train}_k$ and $X^{test}_k$ as the training and test set split of the covariates corresponding with the training and test set split of $\vec {Y} = \{\vec {Y}^{\text {train}}_k, \vec {Y}^{\text {test}}_k \}$ for the *k*th fold. On each training fold, we fit the model and generated posterior samples for each parameter. For each posterior sample *t* after burn-in and thinning, we computed

9 $$\begin{aligned} P(\vec {Y}^{\text {test}}_k| \Theta _o^t, X^{\text {test}}_k) = \prod _{\begin{array}{c} (i,j) \\ \text {uncensored} \end{array}} p(y_{ij}|\Theta _o^t,X_{ij}) \prod _{\begin{array}{c} (i,j) \\ \text {censored} \end{array}} \Pr (y_{ij} > y_{ij}^c \mid \Theta _o^t, X_{ij}, y_{ij}^c) \end{aligned}$$

where $\Theta _o^t$ is a vector of all the *t*th iteration posterior samples for the parameters of the probability distribution of survival based on the *k*th fold of the training data. After computing this quantity for each iteration, we computed an estimate of the out-of-sample posterior predictive likelihood:

10 $$\begin{aligned} \int P(\vec {Y}^{\text {test}}|\Theta _0, X^{\text {test}}) P(\Theta _0|\vec {Y}^{\text {train}},X^{\text {train}})d\Theta _0 \approx \frac{1}{T} \sum _{t=1}^T P(\vec {Y}^{\text {test}}|\Theta ^t, X^{\text {test}}) \end{aligned}$$

where T is the number of sampling iterations after burn-in and thinning. The log-posterior likelihood measures how well a model fits the observed data, with a higher value indicating better fit. After running each model on the training fold and computing the log-posterior predictive likelihood on the corresponding test fold, we took the average of each models’ log-posterior likelihoods to determine which framework provided the best fit.

## Results

### Application to pan-cancer, pan-omics data

Here we describe the application of the hierarchical spike-and-slab model to TCGA data to characterize the clinical relevance of underlying genomic components. To do so, we model patient OS because it is clearly defined, clinically important, and available for most subjects [27]. The model predictors are derived from applying BIDIFAC+ [6] to TCGA pan-omics, pan-cancer data as explained below. Additional methodological details are provided in the “Methods” section and the Additional file

Our data was originally curated for use in Hoadley et al.'s [28] pan-cancer clustering analysis. These data consisted of 29 cancer types and 4 omics platforms. The cancer types are primarily defined by their tissue-of-origin, and we denote each type by its TCGA study abbreviation, e.g., BRCA for breast invasive carcinoma and ESCA for esophageal carcinoma. The omics platforms include (1) RNA-Seq data for 20531 genes, (2) miRNA-Seq data for 743 miRNAs, (3) DNA methylation levels for 22601 CpG sites, and (4) reverse-phase protein array data for 198 proteins. BIDIFAC+ decomposes the data into a sum of low-rank modules, each corresponding to structured variation that exists on a subset of the 4 omics platforms and the 29 cancers. Using this method, [6] identified 50 low-rank modules from which we derived predictors for our model.

We obtained predictors from the BIDIFAC+ results by computing the singular value decomposition (SVD) of each low-rank module to identify underlying components (analogous to principal components) that are specific to a subset of omics platforms and cancer types. For each module’s SVD, we took the product of each singular value with its corresponding right singular vector. This product gives us the component scores for each subject, which will serve as predictors in our survival model. In this context, the BIDIFAC+ components are assumed to be independent and roughly orthogonal. Since BIDIFAC+ can produce components that explain negligible variation in the data, we did not want to consider these as possible predictors in our predictive model. We would not expect these components to explain much variability in OS and they would lead to unnecessary noise. To ensure we consider predictors with the highest likelihood of explaining variation in survival, we developed selection criteria that precedes our modeling step. Our inclusion criteria were as follows:

1. Include the first component from the SVD of each low-rank module. This component explains the most variation within each module.
2. Include any other components whose ratio of eigenvalue (squared singular value) to total variability in the original multi-source, multi-cancer data was greater than 0.01. This amounts to selecting predictors that explain at least 1% of the variation in the original pan-omic, pan-cancer data.

The 0.01 threshold could be adjusted in future studies but it yielded a manageable number of possible model predictors for our purposes. In sum, we considered 66 components derived from the 50 modules as predictors of OS. We refer to each of these predictors by the module from which it was derived and the index of its corresponding right singular vector from the module’s SVD, e.g., predictor 5.1 is the first component from module 5.

To complete our model, we also included a model intercept for each cancer and patient age at the time of diagnosis as a predictor. In our previous work, we showed that age has a strong effect on overall survival in 27 of the 29 cancers considered here [13]. We standardized all predictors to have mean 0 and standard deviation 1 to facilitate comparisons of covariate effects on survival.

We obtained clinical data from the TCGA Clinical Data Resource (TCGA-CDR) [27]. Before running any analyses, we removed subjects who were missing both a survival time and a censoring time, removed subjects who had survival times that were negative or zero, and removed subjects missing a value for age. After filtering, we retained 6856 subjects across 29 cancer types with data from the 4 omics platforms.

We first assessed which of the following model frameworks provided the best predictive performance on the TCGA data factorized by BIDIFAC+. In parentheses, we give the name that each model is henceforth referred to.

1. A hierarchical spike-and-slab model, our proposed model, described in the “Extensions to survival data” section  (“hierarchical”)
2. A model with only a random intercept for each cancer type and no covariates (“null model”)
3. A hierarchical model, with no spike-and-slab component and all covariates are included (“full model”)
4. A hierarchical model with a spike-and-slab component and prior inclusion probabilities fixed at 0.5 [“fixed (0.5)”]
5. A hierarchical spike-and-slab model where a single inclusion probability, $\pi$, is shared for all covariates and all cancer types (as opposed to inferring an inclusion probability, $\pi _\ell$, for each covariate) with a uniform prior $\pi \sim \text{ Beta }(1,1)$ (“shared model”)
6. A joint model in which the proposed hierarchical spike-and-slab model is applied to the 29 cancer types appended together row-wise. This model treats all cancer types as one cancer (“joint model”)
7. The proposed hierarchical spike-and-slab model applied to each of the 29 cancer types separately. The model is fit to each cancer type assuming the cancers are independent of one another (“separate model”)
8. The hierarchical horseshoe accelerated failure time model (“hsaft”) proposed by [14]. We implemented this model using the PanCanVarSel R package provided by the authors [29].

We compared how these models fit the data using 5-fold cross validation of the log-posterior predictive likelihood, as described in the “Posterior predictive likelihood validation” section. After running each model on the training fold and computing the log-posterior predictive likelihood on the corresponding test fold, we took the average of each models’ log-posterior likelihoods to determine which framework provided the best fit.

Our model selection results can be found in Table 1. While the hierarchical and shared model were quite close in fit and predictive accuracy for overall patient survitval, we proceeded with the proposed hierarchical model for the rest of our analysis.

**Table 1:** Model selection results using 5-fold cross validation of the log-posterior likelihood

Model type	Mean log-posterior likelihood
Hierarchical	– 1019.023
Shared	– 1019.968
Fixed (0.5)	– 1036.793
Null model	– 1048.536
Full model	– 1059.670
hsaft	– 1044.561
Separate model	– 1040.328
Joint model	– 1130.240

**Fig. 2:**
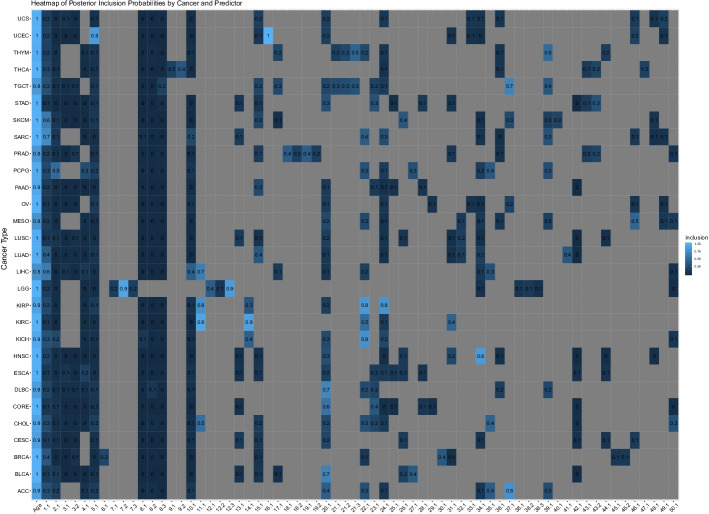
Posterior inclusion probability heatmap for every cancer type and every predictor. The value printed on each box is the posterior probability of inclusion. Gray space indicates a predictor was not available for a particular cancer type. Brighter blue colors indicate higher probability of inclusion, while deeper blue indicators indicate lower probability of inclusion

We ran the hierarchical spike-and-slab model on the factorized TCGA data for 100000 iterations with a 50000 iteration burn-in and 10-iteration thinning. Multiple runs of the model with different initial values gave similar results, suggesting that convergence was satisfactory. We display the variable selection results in Fig. 2 via a heatmap of the posterior inclusion probabilities; an interactive version of this heatmap with links to additional plots displaying componeents with high posterior probabilities is available at www.ericfrazerlock.com/PanTCGA_Inclusion_Heatmap.pdf. The posterior inclusion probability for covariate $\ell$ in cancer *i* is the average of its inclusion indicators generated by our model after burn-in and thinning. Age was included for every cancer type with uniformly high probability, while the inclusion of pan-omic components were comparatively sparse. BIDIFAC+ predictors that capture molecular variation across all or most cancer types were mostly not included by our model. However, certain BIDIFAC+ predictors were identified as predictive of patient survival with high probability and are summarized in Table 2, ordered by descending posterior inclusion probability. In total, our hierarchical spike-and-slab model selected 24 BIDIFAC+ components across 17 cancer types, based on a posterior inclusion probability above 0.5.

**Table 2:** Variable selection results from hierarchical spike-and-slab model. The “Component” column gives the module and component number that was selected, the “Cancer” column gives the cancer for which it was selected, “Mean Effect” gives the mean posterior draw for the coefficient of the selected covariate, “Posterior Inclusion Probability” gives the average of inclusion indicators, and “Credible Interval” gives 95% credible interval for coefficient effect

	Component	Cancer	Mean effect	Credible interval	Posterior inclusion probability
1	16.1	UCEC	– 0.50	(– 0.723, – 0.273)	1.00
2	11.1	KIRC	0.36	(– 0.004, 0.678)	0.92
3	7.2	LGG	– 0.47	(– 0.773, 0.004)	0.92
4	14.1	KIRC	– 0.30	(– 0.59, 0.005)	0.88
5	12.3	LGG	– 0.29	(– 0.551, 0.008)	0.86
6	5.1	UCEC	– 0.35	(– 0.62, 0.006)	0.86
7	34.1	HNSC	– 0.33	(– 0.599, 0.007)	0.84
8	22.1	KIRP	0.77	(– 0.011, 1.435)	0.82
9	24.1	KIRP	– 0.62	(– 1.218, 0.012)	0.79
10	37.1	ACC	0.54	(– 0.013, 1.228)	0.79
11	22.1	KICH	0.53	(– 0.012, 1.191)	0.78
12	11.1	KIRP	0.36	(– 0.016, 0.964)	0.77
13	1.1	SARC	0.30	(– 0.012, 0.662)	0.74
14	20.1	DLBC	0.54	(– 0.017, 1.628)	0.70
15	37.1	TGCT	0.49	(– 0.017, 1.436)	0.68
16	20.1	BLCA	– 0.21	(– 0.516, 0.012)	0.68
17	11.1	LIHC	0.21	(– 0.079, 0.646)	0.66
18	1.1	LIHC	0.25	(– 0.014, 0.659)	0.63
19	1.1	SKCM	0.18	(– 0.014, 0.473)	0.63
20	20.1	CORE	– 0.15	(– 0.434, 0.015)	0.57
21	11.1	CHOL	0.09	(– 0.34, 0.673)	0.54
22	22.1	KIRC	0.12	(– 0.015, 0.452)	0.51
23	39.1	THYM	– 0.28	(– 1.052, 0.018)	0.51
24	2.1	PCPG	0.45	(– 0.016, 1.642)	0.50

Note that the sign of the effects and credible intervals are not immediately interpretable because the identified components (given by singular vectors of an SVD) are uniquely defined up to their sign. However, we can interpret the scale of the effect. Motivated by these results, we chose to investigate more deeply components included for uterine corpus endometrial carcinoma (UCEC), brain lower grade glioma (LGG), kidney renal papillary cell carcinoma (KIRP), kidney renal clear cell carcinoma (KIRC), and kidney chromophobe (KICH) to understand the clinical relevance of the pan-omic components selected. In what follows, we describe our investigation into the clinical significance of these components. While the figures and discussion describe the marginal effects of components, bear in mind that because our model is multivariate the identification of a component’s predictive power for survival is relative to the information contained in other model predictors. We describe here our investigation into components 16.1 for UCEC, 7.2 for LGG, and 11.1 for KIRP and KIRC; we explore the inclusion of additional components for UCEC, LGG, and KICH in in the Additional file 1: Appendix B.

**Fig. 3:**
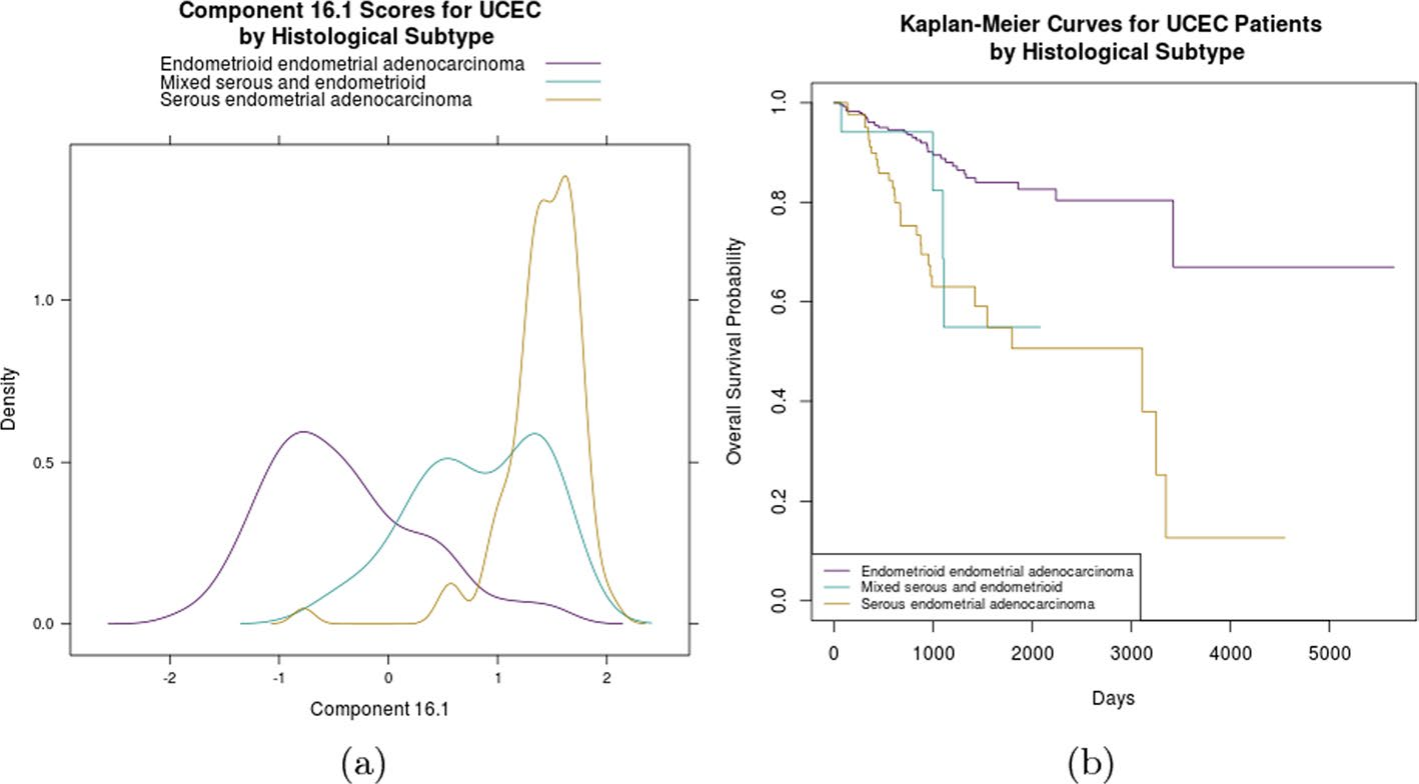
Figure 3**a** displays a KDE plot for the selected component 16.1, which was identified as predictive of survival in UCEC subjects. Component 16.1 scores for subjects with serous UCEC cluster separately from subjects with endometrioid and mixed UCEC. Figure 3**b** shows the Kaplan–Meier survival curves for each of the histological subtypes, with the serous subtype showing the worst survival

BIDIFAC+ component 16.1 was identified as predictive of survival by the model with near certainty (Table 2). We investigated if this component was associated with UCEC’s three histological subtypes: endometrioid, serous, and mixed serous and endometroid [30]. We examined this using histological labels provided in TCGA-CDR [27]. Based on the kernel density estimation (KDE) graph shown in Fig. 3a, the three UCEC histological subtypes cluster distinctly along component 16.1. This suggests that this pattern of variation is primarily driven by distinctions between the three types of UCEC tumors. The Kaplan–Meier survival figure provided in Fig. 3b shows divergent survival outcomes for the three subtypes. [30] found that serous and serous-like tumors show extensive somatic copy number alterations (SCNAs), while endometrioid tumors do to a lesser degree, and observed that SCNAs roughly correlated with progression-free survival. While we modeled OS, this may be an underlying latent variable.

**Fig. 4:**
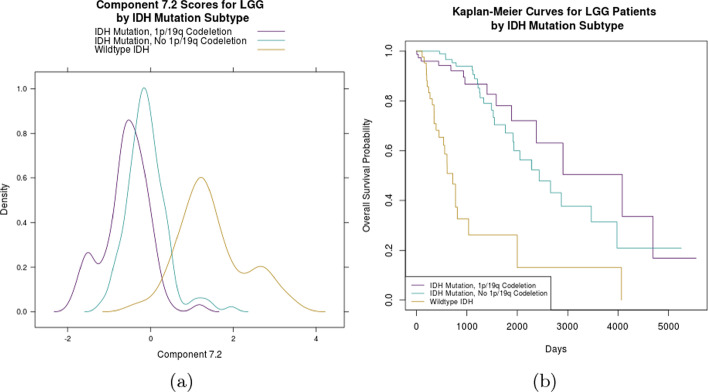
Figure 4**a** displays a KDE plot for the selected component 7.2, which was identified as predictive of survival in LGG patients. Estimated distributions are colored by IDH mutation and 1p/19q codeletion status. Figure 4**b** shows the Kaplan–Meier survival curves for each of the mutation subgroups, with the IDH wildtype mutation showing worst overall survival

BIDIFAC+ component 7.2 was identified as predictive of survival in LGG subjects with a posterior probability of 0.92. We considered its association with the mutation status of genes IDH1 and IDH2 and deletion status in chromosome arms 1p and 19q (1p/19q codeletion) using data from [31]. Mutations in these genes define most cases of LGG and contribute to an LGG subtype associated with better survival [31]. We saw subjects with wild-type IDH mutation clustered distinctly along component 7.2 (Fig. 4a), with Kaplan–Meier survival curves in Fig. 4b displaying divergent survival patterns among the three IDH mutation groups. This suggests that this pattern of variation is linked to IDH mutation patterns that correlate with patient survival.

**Fig. 5:**
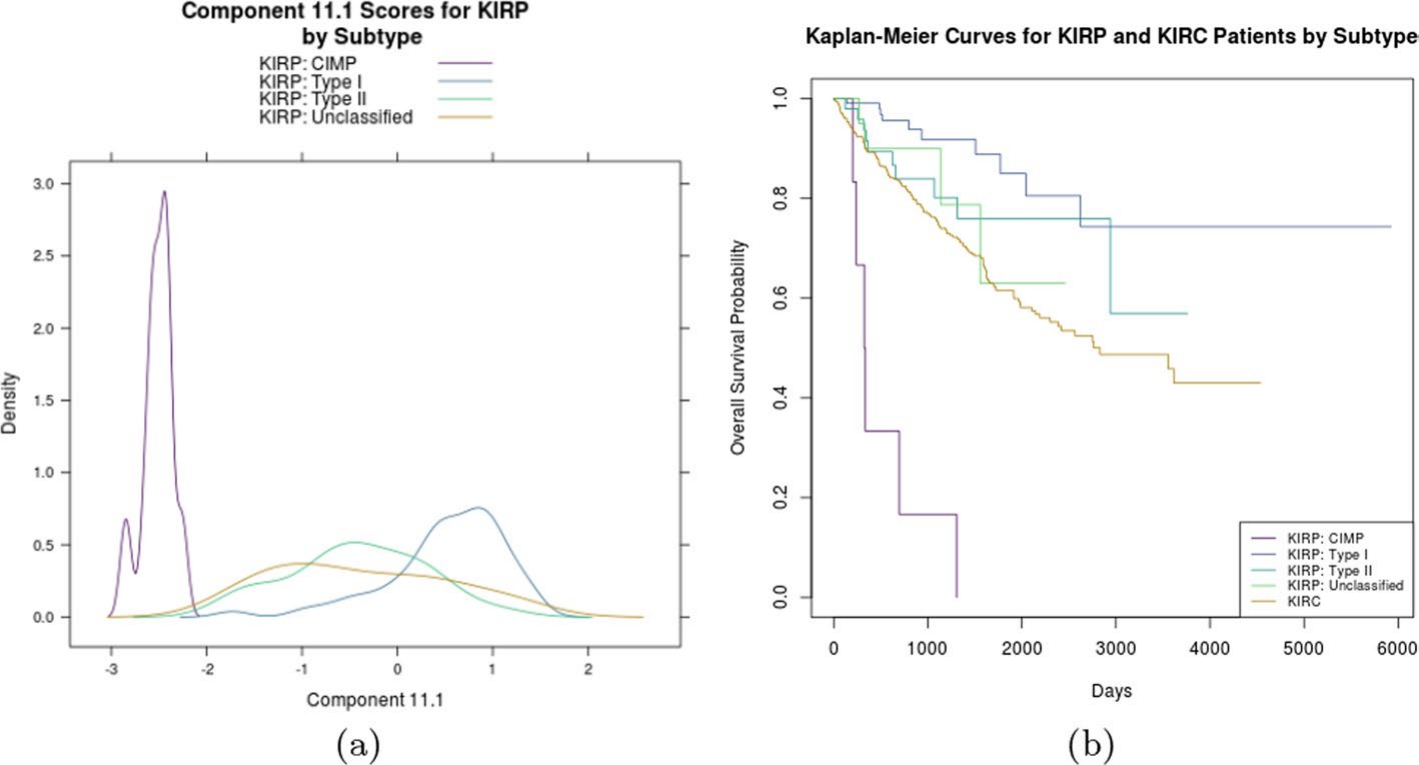
Figure 5**a** displays a KDE plot for component 11.1 within each of the KIRP subtypes, showing CIMP subjects clustering distinctly along component 11.1. This pattern of variation was identified as predictive of survival in KIRP as well as KIRC subjects. Figure 5**b** shows a Kaplan–Meier survival plot for all KIRP and KIRC subjects, showing KIRP subjects with the CIMP subtype have the poorest survival. Though KIRC does not currently have a known CIMP subtype, the clinical significance of this subtype is well-known for KIRP. Our analysis suggests a similar distinction may exist in KIRC that is also clinically relevant

Lastly, component 11.1 was associated with survival for KIRP and KIRC subjects. Using the classification scheme from TCGA’s pan-renal project, samples were documented as either KIRP or KIRC, with KIRP further subdivided into type I, type II, and CIMP. Any KIRP patients who did not fit into these categories were left unclassified. CIMP refers to a CpG island methylator phenotype [32] and type I and type II are characterized by specific genetic mutations [33]. The CIMP subgroup is known to have the poorest survival of all renal cancers [32], which prompted us to examine if this pattern of variation captured this distinction using data from [32]. Figure 5a shows KIRP subjects classified as CIMP cluster separately from the other three subgroups. Kaplan–Meier survival curves emphasize the stark survival difference between CIMP and the remaining KIRP and KIRC subjects. KIRC also shows poorer survival compared to KIRP types I, II, and unclassified subjects (Fig. 5b). While it seems that 11.1 is associated with KIRP clinical subtypes (e.g., CIMP), it is unclear to what characteristics of KIRC these predictors are linked. The presence and clinical relevance of the CIMP phenotype has been well-studied for KIRP, and our analysis suggests that similar distinctions exist within KIRC that are also clinically relevant.

### Simulation study

We now present a simulation study to compare different approaches to hierarchical variable selection with the spike-and-slab prior. The primary goal of our simulation study was to characterize how modifications to the hierarchical variable selection component of our proposed model perform under different data-generating schemes, specifically in the context of our data application. We compared our proposed model under various data-generating schemes to the seven other modeling frameworks considered in the “Application to pan-cancer, pan-omics data” section.

We designed the data-generating schemes to mimic our TCGA data application in the “Application to pan-cancer, pan-omics data” section by generating groups of the same sample size, with the same number of covariates for each group, and by randomly right-censoring subjects. The degree of overlap across groups for each covariate matches that in our data application, which allows the possibility that some covariates are shared across all groups, some covariates are shared across subsets of groups, and some covariates are present in only one group. Each model assumed a log-normal outcome and approximately 50% of subjects were censored.

We considered six data-generating scenarios:

1. Each covariate is included for all groups for which it is available with probability 0.5 or excluded for all groups for which it is available with probability 0.5
2. Each covariate is included for all groups for which it is available with probability 0.1 or excluded for all groups for which it is available with probability 0.9
3. Each covariate is included independently for each group with probability 0.5, i.e. no true hierarchical structure
4. Each covariate is included independently for each group with probability 0.1
5. All covariates are included in the model
6. All covariates are excluded in the model

We compared the performance of the eight models using two metrics: the mean sum-of-squared deviations (SSD) between the true inclusion indicator and the posterior inclusion estimated by each model and the log-posterior predictive likelihood defined in the “Posterior predictive likelihood validation” section. The mean SSD provides a measure of selection accuracy while the log-posterior predictive likelihood provides a measure of predictive accuracy.

We now define the mean sum-of-squared deviations. Assume the true inclusion indicator for covariates $\ell$ available for group *i* to be $\gamma _{i\ell }$. Let $\hat{\gamma }_{i\ell }$ be the posterior inclusion probability for the covariates of group *i* estimated by the model; $\hat{\gamma }_{i\ell }$ is computed by averaging the inclusion indicators from each model iteration after burn-in and thinning. The mean SSD for model *k*, $k=1,\dots , 8$ is

11 $$\begin{aligned} SSD_{k} = \frac{1}{M}\sum _{i=1}^{29} \sum _{l \in S_i} (\gamma _{i \ell } - \hat{\gamma }_{i\ell })^{2} \end{aligned}$$

where $M = \sum _{i=1}^{29} |S_i|$ is the total number of regression coefficients in the model. A lower mean SSD reflects better variable selection accuracy, while a higher mean SSD reflects poorer variable selection accuracy. We averaged mean SSDs across simulation replications. We do not compute the SSD metric for the hsaft model because the horseshoe prior does not inherently model a probability that a given predictor is included or excluded.

To compare all eight models based on their log-posterior predictive likelihoods, we generated a training data set under each data-generating condition. After fitting the model on this training data set, we computed the log-posterior likelihood on a test data set which is generated under the same condition and with the same true parameter values as the training data set. We averaged the resulting log-posterior likelihoods across simulation replications.

We designed our simulation as follows. For 100 replications,

1. Run each of the eight considered models under the six data-generating conditions
2. For each condition, generate the data accordingly and run each model for 10000 iterations
3. After a 5000 iteration burn-in and 10-iteration thinning, compute the mean SSD
4. Generate a test data set under the same data-generating conditions. Using the posteriors samples generated based on the training data in step 3, compute the log-posterior predictive likelihood
5. At the end of the simulation, average across all simulation replications

We used 100 replications to ensure consistent results when the simulation study was repeated. The resulting mean SSDs and log-posterior likelihoods are shown in the Tables 3 and 4, respectively, where we bold the best performing model. In both tables, we use pairwise t-tests as a simple way to assess whether the observed differences in performance across the simulation replications are statistically significant. If the performance of two models were not significantly different under a particular data-generating condition, they were considered to have performed equally well.

**Table 3:** Mean sum of squared deviations for each model under each data-generating condition. Row names correspond to the pattern of covariate inclusion. Column names correspond to the models. Bolded values indicate the best performing model based on a pairwise t-test. If multiple values are bolded, then model performances were not significantly different at 0.01 level

	Hierarchical	Fixed (0.5)	Full model	Shared	Null model	Joint	Separate
All In (Prob = 0.5)	**0.0307**	0.1124	0.4927	0.1507	0.5073	0.0644	0.0975
All In (Prob = 0.1)	**0.0251**	0.1204	0.8990	0.0411	0.1010	0.0314	0.0796
Indep. In (Prob = 0.5)	0.0915	**0.0854**	0.5000	**0.0854**	0.5000	0.4002	0.0977
Indep. In (Prob = 0.1)	0.0366	0.0813	0.8987	**0.0269**	0.1013	0.2424	0.0625
All In	0.0529	0.1304	**0.0000**	0.0016	1.0000	0.0981	0.1431
None In	0.0162	0.1103	1.0000	0.0002	**0.0000**	0.0211	0.0637

Bold values indicate the best performing model for each scenario, and multiple values are bolded if they are not significantly different from the best model at the 0.01 level

**Table 4:** Mean log-posterior predictive likelihood for each model under each data-generated condition. Row names correspond to the pattern of covariate inclusion. Column names correspond to the models. Bolded values indicate the best performing model based on a pairwise t-test. If multiple values are bolded, then model performances were not significantly different at 0.01 level

	Hierarchical	Fixed (0.5)	Full model	Shared	Null model	Joint	Separate	Hsaft
All In (Prob = 0.5)	**– 7687.97**	– 7771.80	– 7883.81	– 7783.47	– 11662.95	– 10675.67	– 7819.95	– 7893.56
All In (Prob = 0.1)	**– 8221.97**	– 8310.11	– 8520.15	– 8229.85	– 9729.56	– 9869.96	– 8336.16	– 8359.26
Indep. In (Prob = 0.5)	**– 7755.32**	**– 7751.48**	– 7951.62	**– 7749.98**	– 11701.51	– 11287.71	– 7815.04	– 7908.40
Indep. In (Prob = 0.1)	– 7360.53	– 7421.77	– 7723.09	**– 7349.98**	– 9580.55	– 10358.29	– 7447.81	– 7488.49
All In	– 307.15	– 8359.16	**– 8291.82**	– 8292.16	– 12863.43	– 11096.83	– 8436.40	– 8409.30
None In	– 7827.13	– 7918.48	– 8173.49	– 7804.41	**– 7800.81**	– 9504.38	– 7935.25	– 7921.88

Bold values indicate the best performing model for each scenario, and multiple values are bolded if they are not significantly different from the best model at the 0.01 level

The proposed hierarchical spike-and-slab model performs best under conditions (1) and (2) because each group affords concordant information about each predictor. In this case, it is beneficial to borrow strength across groups when estimating the prior inclusion probability. Unlike conditions (1) and (2), conditions (3) through (6) are not specifically suited to the proposed model but competitive mean SSDs and posterior likelihoods demonstrate its competitive performance. Under condition (3), when the prior inclusion probability was 0.5 for all covariates, the fixed (0.5) model naturally performs best. The shared model performs well under both (3) and (4) because there was no added benefit to estimating the prior inclusion probability separately for each covariate. Conditions (5) and (6) were the most extreme, under which all or none of the covariates were included, respectively. Under (5) and (6), the full model (a hierarchical model without a spike-and-slab component) and the null model perform best, respectively. Like (3) and (4), the shared model is competitive because there was no added advantage to estimating a prior inclusion probability for each covariate; however, our proposed model remained comparable in selection and prediction accuracy. Under conditions (1), (2), (5), and (6), the joint model performs variable selection comparably well because the groups do not differ in their inclusion patterns; however, its predictive performance across all conditions is starkly poorer. The hsaft model offers relatively competitive predictive performance under all conditions, despite the generative model not matching its assumptions.

This simulation study demonstrates that the hierarchical spike-and-slab prior described in the “Methods” section is competitive in its ability to correctly identify which covariates to include under the data-generating scenarios considered here. While it offers flexibility to estimate different inclusion probabilities for each covariate and pool information across groups, it can also perform well when borrowing strength is not advantageous. Under each condition, the proposed model fit the test data well, exhibiting its strength in identifying a model with predictive power. While it may not be the optimal model under each condition, its performance was consistent with models that were tailored to perform well, making it a flexible option for an array of underlying data-generating mechanisms.

## Discussion

In this article, we address prediction across multiple sources of data and multiple samples sets by using molecular patterns of variability identified by BIDIFAC+ in a Bayesian hierarchical model. Our model uses spike-and-slab priors that borrow information across groups in determining the model’s sparsity structure. This spike-and-slab framework has the advantage of increasing power to detect covariate inclusion and covariate effect while being flexible enough to allow each group to have a different covariate set. It also induces correlation under the posterior between selected predictors. An additional perk of this prior is the natural “inclusion/exclusion” interpretation that other shrinkage methods, like the horseshoe prior [20] or the Bayesian lasso [18], lack.

We apply this model to TCGA data where we use BIDIFAC+ components from the integration of pan-omics, pan-cancer data as predictors for overall patient survival. Predictive modeling using these data expands upon the exploratory work of [6] and contributes to the body of research in prediction using multi-source, multi-sample set data. Factorizing the pan-omic, pan-cancer data prior to predictive modeling is useful because the original genomic data is very high dimensional which presents issues of multicollinearity for modeling. Our model gave sparse results regarding selected predictors that explain variability across a large number of cancers. However, it did identify several molecular patterns within smaller subsets of cancer types that are strongly informative of survival, including clinically relevant molecular distinctions that have been previously established (e.g., subtypes within UCEC and LGG) and similar effects across cancer types that warrant further investigation (e.g., for the kidney cancers). In our context, we assumed BIDIFAC+ components were independent and orthogonal; however, this approach could be extended to incorporate correlation in the components if a correlation structure is known a priori. Other worthwhile future directions include considering different parametric assumptions for the survival model and relaxing the assumption that the error variance is shared across groups. Additionally one could consider non-linear models and generalized additive models in this context. Including other clinical covariates, like stage and grade, might elucidate the effect of BIDIFAC+ predictors on overall survival; however, stage and grade are not uniformly defined over different cancer types, which presents a challenge for their use in pan-cancer clinical modeling. Alternatively, one may consider other clinical variables as the response, like progression-free survival, but the availability of such data is not as widespread for the TCGA cohort [27]. Patterns of variation identified by BIDIFAC+ on other omics sources, such as copy number variation, could also be considered as predictors.

We also present results from a simulation study where we evaluate the performance of modifications to the variable selection component in our proposed Bayesian model in our data application context. The goal of this study was to characterize the flexibility of our hierarchical spike-and-slab prior in fitting a diverse array of data-generating schemes that mimic our application’s group structure. Our simulation study showed that this prior was competitive under all six data-generating conditions considered. This study could be expanded to compare the proposed model to other survival models, such as the proportional hazards model or models assuming different parametric survival distributions. Incorporating other Bayesian variable selection methods, like the horseshoe prior [20] and the Bayesian lasso [18], into these models would also be worthwhile for comparison.

For our TCGA application, we fit our model more than once from random starting values and observed consistent results across these randomly-initialized chains, suggesting that the sampler converges and appropriately covers models with high posterior probability. However, for other scenarios a potential drawback of the spike-and-slab approach is that in the presence of many predictors, the posterior sampling algorithm may not cover all possible models. Another potential drawback of our approach is that it does not explicitly incorporate biological relationships between the omics features that are known *a priori*, and the associations captured are not necessarily causal. In general, more work can be devoted to devising variable selection methods that borrow information across grouped data. It would be valuable to evaluate and compare the performance of these extensions, in addition to the spike-and-slab model we discuss here, to characterize their relative advantages and disadvantages in a hierarchical setting.

## Conclusion

We expand upon the exploratory results of bidimensional integration of multiple sources of data and multiple sample sets by using BIDIFAC+ components in a Bayesian hierarchical survival model with spike-and-slab priors. Our results show that molecular patterns of variability identified by BIDIFAC+ were predictive of survival in subsets of cancers available in TCGA and may offer insight into novel clinical subtypes. We also show that the spike-and-slab prior is a suitable option for Bayesian variable selection on grouped data in several different data-generating scenarios.

## Additional file

**Additional file 1.** This supplementary document provides additional details on the Gibbs sampling algorithm for posterior computation, further details on the TCGA data application, and an additional simulation study to validate the model and sampling algorithm.

## Data Availability

The code and data generated and analysed during the current study are available in the HierarchicalSS_PanCanOmics repository, https://github.com/sarahsamorodnitsky/HierarchicalSS_PanCanPanOmics/.

## Abbreviations

BIDIFAC: Bidimensional integrative factorization
TCGA: The Cancer Genome Atlas
mRNA: Messenger ribonucleic acid
miRNA: Micro ribonucleic acid
PCA: Principal components analysis
JIVE: Joint and individual variance explained
SLIDE: Structural learning and integrative decomposition
GIPCA: Generalized integrative principal components analysis
OS: Overall survival
BRCA: Breast cancer invasive carcinoma
ESCA: Esophageal carcinoma
DNA: Deoxyribonucleic acid
SVD: Singular value decomposition
TCGA-CDR: TCGA Clinical Data Resource
hsaft: Horseshoe shrinkage accelerated failure time
UCEC: Uterine corpus endometrial carcinoma
LGG: Brain lower grade glioma
KIRP: Kidney renal papillary cell carcinoma
KIRC: Kidney renal clear cell carcinoma
KICH: Kidney chromophobe
SSD: Sum of squared deviations
CpG: Cytosine-phosphate-guanine
CIMP: CpG island methylator phenotype
