# Supplementary material for: A hierarchical spike-and-slab model for pan-cancer survival using pan-omic data
Source: BMC Bioinformatics. 2022 Jun 17;23:235. doi: 10.1186/s12859-022-04770-3 (PMC9204947; doi:10.1186/s12859-022-04770-3)
Supplement: Supplementary file 1 — Additional file 1. This supplementary document provides additional details on the Gibbs sampling algorithm for posterior computation, further details on the TCGA data application, and an additional simulation study to validate the model and sampling algorithm. [file 12859_2022_4770_MOESM1_ESM.pdf]

RESEARCH

A Hierarchical Spike-and-Slab Model for Pan-Cancer Survival Using Pan-Omic Data: Appendix and Supplementary Materials

Sarah Samorodnitsky[1], Katherine A. Hoadley[2] and Eric F. Lock[1*]

*Correspondence: elock@umn.edu
[1]Division of Biostatistics, University of Minnesota, Minneapolis, USA
Full list of author information is available at the end of the article

Appendix A: Model Fitting Algorithm Details

We used an in-house Gibbs sampler to estimate the parameters of our model. At each iteration of the algorithm, we generated a sample of each parameter from its respective conditional posterior distribution. In this section, we outline the conditional posterior of each parameter in our model.

The conditional posterior of π_ℓ for the ℓth covariate is:

$$\pi_\ell|\gamma_{.\ell} \sim \text{Beta}\left(1 + \sum_{j=1}^{T_\ell} \gamma_{j\ell}, \;\; 1 + T_\ell - \sum_{j=1}^{T_\ell} \gamma_{j\ell}\right)$$

where $\gamma_{.\ell}$ represents the vector of inclusion indicators for the ℓth covariate for all 29 cancer types and T_ℓ is the number of cancer types that for which the ℓth covariate is available.

The conditional posterior for $\gamma_{i\ell}$, the inclusion indicator for the ith cancer type and ℓth covariate is:

$$P(\gamma_{i\ell} = 1 \mid \beta_{i\ell}, \tilde{\beta}_\ell, \lambda_\ell^2) = \frac{\pi_\ell \text{N}(\beta_{i\ell}; \tilde{\beta}_\ell, \lambda_\ell^2)}{\pi_\ell \text{N}(\beta_{i\ell}; \tilde{\beta}_\ell, \lambda_\ell^2) + (1-\pi_\ell) N(\beta_{i\ell}; 0, \frac{1}{10000})}$$

where $N(\cdot, \cdot)$ refers to the density of a normal distribution.

The vector of coefficients for the ith cancer type, $\beta_{i.}$ has the following conditional posterior:

$$\beta_{i.} \mid \{X_i, Y_i, \gamma_{i.}, \sigma^2, \lambda^2, \tilde{\beta}\} \sim \text{Normal}(Bb, B)$$

where

$$B = \left[\frac{1}{\sigma^2} X_i^T X_i + \Sigma_i^{-1}\right]^{-1}$$

and

$$b = \frac{1}{\sigma^2} X_i^T y_i + \Sigma^{-1} diag(\gamma_{i.})\tilde{\beta}$$

based on the results of [1]. Here, X_i represents the covariate set for group i. y_i represents the outcome vector for group i. $\gamma_{i.}$ represents the vector of inclusion indicators for group i.

The conditional posterior for $\tilde{\beta}_\ell$ is:

$$\tilde{\beta}_\ell \mid \{\beta_{.\ell}, \lambda_\ell^2\} \sim \text{Normal}\left(\frac{K_\ell \tau^2 \bar{\beta}_\ell}{\lambda_\ell^2 + K_\ell \tau^2}, \frac{\lambda_\ell^2 \tau^2}{\lambda_\ell^2 + K_\ell \tau^2}\right)$$

where $K_0 = 29$ because the model for every cancer type has an intercept, K_ℓ for $\ell = 1, \ldots, 67$ is the number of cancer types that have this covariate and are not in the spike, and $\bar{\beta}_\ell = \frac{1}{K_\ell}\sum_{i=1}^{K_\ell} \beta_{i\ell}$. τ^2 is fixed at 10^2 for $\ell = 0$ and 1 for $\ell = 1, \ldots, 67$. Note that 67 is the number of BIDIFAC+ components in our model plus age.

The conditional posterior for λ_0^2 is:

$$\lambda_0^2 \mid \{\beta_{.0}, \tilde{\beta}_0\} \sim \text{Inverse-Gamma}\left(\frac{K_0}{2} + 1, 1 + 0.5W_0\right)$$

where $K_0 = 29$ because the model for every cancer type has an intercept and $W_0 = \sum_{i=1}^{K_0}(\beta_{i0} - \tilde{\beta}_0)^2$.

The conditional posterior for λ_ℓ^2, for $\ell = 1, \ldots, 67$ is:

$$\lambda_\ell^2 \mid \{\beta_{.\ell}, \tilde{\beta}_\ell\} \sim \text{Inverse-Gamma}\left(\frac{K_\ell}{2} + 5, 1 + 0.5W_\ell\right)$$

where K_ℓ is the number of cancer types for which this covariate is available and also is not in the spike and $W_\ell = \sum_{i=1}^{K_\ell}(\beta_{i\ell} - \tilde{\beta}_\ell)^2$. W_ℓ only includes the $\beta_{i\ell}$ which are not in the spike.

The conditional posterior for σ^2 is:

$$\sigma^2 \mid \{X, Y\} \sim \text{Inverse-Gamma}\left(\frac{N}{2} + 0.01, \frac{1}{2}B + 0.01\right)$$

where N is the number of observations in the model and $B = \sum_{i=1}^{29}(y_i - X_i\beta_{i.})^2$.

Appendix B: Further Details on Data Application Results

Here we provide additional figures and discussion of the results for our application of TCGA data, expanding on our summary in the main article.

Two BIDIFAC+ components were identified as predictive of survival for UCEC subjects: 16.1 and 5.1. We observed that component 16.1 differentiated UCEC subjects according to their histological subtype: endometrioid, serous, and mixed histology; however, the link between variability explained by component 5.1 and clinical features of UCEC appeared less obvious. Figure 1a shows the histological subtypes of UCEC are not well differentiated by component 5.1. Figure 1b shows how subjects cluster along components 16.1 and 5.1 according to subtype, offering a comparison between the variation explained by either component. However, this pattern of variation characterized by component 5.1 does have a marginal association with survival. This can be observed from the Kaplan-Meier survival plot in figure 1c,

which shows how survival outcomes differ by subjects depending on the sign of their scores for this component.

Two BIDIFAC+ components were identified as predictive of survival in LGG, components 7.2 and 12.3. We considered the association of these components with mutation status of genes IDH1 and IDH2 and found that component 7.2 differentiates three types of IDH mutations. These mutation groups also displayed starkly different survival outcomes, with IDH wildtype mutations showing worst overall survival. Component 12.3 appeared to differentiate the IDH mutation subgroups to a lesser degree, with IDH wildtype mutations showing more variation across subjects than either of the other two groups, as shown in figure 2a. Figure 2b demonstrates how subjects cluster when plotted against component 7.2 and 12.3, allowing a comparison of how much variation each component explains. The pattern of variability characterized by component 12.3 does have a marginal association with survival, as shown by the Kaplan-Meier survival figure in figure 2c, which shows how survival outcomes differ by subjects depending on the sign of their component 12.3 scores.

Lastly, we considered the components identified as predictive of survival in KIRP, KIRC, and KICH. BIDIFAC+ component 11.1 was selected for both KIRP and KIRC and component 22.1 was selected for all three. In our main article, we saw KIRP subjects grouped by subtype cluster along component 11.1, with the CIMP methylator phenotype clustering very distinctly from the rest. This subgroup also showed the worst overall survival of all KIRP subtypes. Since this pattern of variability also appears to explain variation in survival outcomes in KIRC subjects, we suggested a similar CIMP phenotype may be present in KIRC, though such a subtype is not currently defined for this cancer.

Component 22.1 appeared to distinguish the CIMP methylator phenotype in KIRP to a lesser degree. This can be observed in the KDE figure and scatterplot of KIRP subjects along components 11.1 and 22.1 in figures 3a and 3b. The inclusion of 22.1 for KIRC and KICH remains unclear, warranting further investigation as these are cancers that are not currently differentiated into subtypes. It seems this pattern of variation is some feature that is not directly related to histological subtypes. Nevertheless, component 22.1 is marginally associated with survival in both KICH and KIRC. This is shown in figure 3c, where subjects with positive and negative scores for component 22.1 have distinct survival outcomes.

Appendix C: Validation Study

In addition to the large-scale simulation comparison described in Section 2.2 of the main article, we ran two simulations to validate our Gibbs sampling algorithm and spike-and-slab model. The first simulation checked the coverage rates of credible intervals based on posteriors draws from our in-house Gibbs sampler. For each iteration of this simulation, we generated true values for each parameter from their respective priors, including inclusion indicators for the spike-and-slab component. These were then used to generate simulated data. Using these data, we generated posterior draws from our Gibbs sampler, calculated 95% credible intervals, and checked whether the true value was indeed captured by the credible interval. The entire simulation is outlined below:

1 For $iter = 1, \ldots, 1000$,

(a) Fix the number of clusters at a chosen value. Randomly generate sample sizes for each cluster from 50 to 500.
(b) Generate predictors from a Normal$(0, 1)$ to be stored in matrix $\mathbf{X}$ and generate true values for each of the model parameters from their respective priors. The true values are denoted as vectors β^*, $\tilde{\beta}^*$, λ^{2*}, γ^* , and σ^{2*}. Based on the inclusion indicators, γ^*, the excluded β^* will either be generated from the spike or the slab distribution.
(c) Generate survival times from a normal distribution with mean $\mathbf{X}\beta^*$ and variance σ^{2*}. Use this distribution to generate censor times. Replace survival times with censor times if the survival time is greater than the corresponding censor time with "not available" (NA).
(d) Run the Gibbs sampler based on the generated data for 2000 iterations. Use a 1000 iteration burn-in and compute 95% credible intervals.
(e) Compare the resulting credible intervals with the generated true values. Tally how many times the credible intervals contained the true parameters.

2 Check that for approximately 95% of iterations, the true value was contained in its respective credible interval.

We set up a second simulation to assess the accuracy of the spike-and-slab component of the model. We followed an identical set up to the one above but instead of considering 95% credible intervals, we calculated the posterior inclusion probability for each predictor by averaging the number of times a covariate was included after burn-in. If this mean was greater than 0.5, we considered that covariate as "included," otherwise it was considered "excluded." We then compared this posterior inclusion indicator with the true inclusion indicator and tallied the proportion of times our model correctly identified a predictor should be included. The design of this simulation is described in more detail below:

1 For $i = 1, \ldots, 1000$:
(a) Follow the same strategy as in the previous simulation to generate data.
(b) Run the Gibbs sampler on the simulated data.
(c) Store the inclusion/exclusion results from the sampler after including a burn-in. If a covariate was included over 50% of the time, the covariate was considered "included." Otherwise, it was considered "excluded."
(d) Compare the inclusion/exclusion results from the Gibbs sampler with the true inclusion/exclusion indicators. Tally the number of times the model correctly identified a covariate as included or excluded.

2 Confirm nominal accuracy of the model. We saw our model correctly identified predictors approximately 90% of the time.

In both validation studies, we assumed there were 12 clusters. The model for each cluster contained an intercept, as well as a subset of three possible covariates. Similar to our TCGA data application, we assumed the clusters did not share identical covariate sets. We generated outcomes for each cluster from a normal distribution and censored approximately 50% of subjects. We assigned the following priors for each of the model parameters (and these were the priors from which we generated the true values for this simulation in step 1b):

For $i = 1 \ldots, 12$, let $S_i = \{\ell : X_\ell \text{ is a predictor for cluster } i\}$. Assume a linear model for the jth response in cluster i:

$$y_{ij} = \beta_{i0} + \sum_{\ell \in S_i} \beta_{i\ell} X_{ij\ell} + \epsilon_{ij}$$

where $\epsilon_{ij} \sim N(0, \sigma^2)$. We excluded the intercept from the spike-and-slab framework and assumed $\beta_{i0} \sim \text{Normal}(\tilde{\beta}_0, \lambda_0^2)$. For the remaining predictors, $j \in S_i$:

$$\beta_{ij} \sim (1 - \gamma_{ij})\text{Normal}\left(0, \frac{1}{10000}\right) + \gamma_{ij}\text{Normal}(\tilde{\beta}_k, \lambda_j^2)$$

Assume $\tilde{\beta}_0 \sim \text{Normal}(0, 10^2)$ and $\lambda_0^2 \sim \text{Inverse-Gamma}(1, 1)$. Further assume $\tilde{\beta}_\ell \sim \text{Normal}(0, 1)$ and $\lambda_\ell^2 \sim \text{Inverse-Gamma}(5, 1)$ for $j = 1, 2, 3$. Lastly, we used assume the variance in the survival outcomes $\sigma^2 \sim \text{Inverse-Gamma}(1, 1)$. Our priors and likelihood differed slightly from those used in our analysis for computational ease.

The coverage results for this simulation can be found in table 1, which shows coverage rates for each parameter and covariate combination. The selection accuracy results can be found in table 2. These results demonstrate nominal coverage rates which confirms the model is running properly.

Author details
[1]Division of Biostatistics, University of Minnesota, Minneapolis, USA. [2]Department of Genetics, Lineberger Comprehensive Cancer Center, University of North Carolina at Chapel Hill, Chapel Hill, USA.

References
1. Lindley, D.V., Smith, A.F.M.: Bayes estimates for the linear model. Journal of the Royal Statistical Society. Series B (Methodological) **34**(1), 1–41 (1972)

Parameter	Intercept $j=0$	Covariate $j=1$	Covariate $j=2$	Covariate $j=3$
$\tilde{\beta}$	0.944	0.950	0.961	0.939
λ^2	0.952	0.957	0.949	0.958
π		0.925	0.938	0.953
$\beta_{1.}$	0.951	0.928		0.951
$\beta_{2.}$	0.937	0.948	0.943	0.955
$\beta_{3.}$	0.941		0.948	
$\beta_{4.}$	0.948	0.937	0.937	
$\beta_{5.}$	0.947			0.929
$\beta_{6.}$	0.959	0.946		0.942
$\beta_{7.}$	0.948		0.940	
$\beta_{8.}$	0.944	0.943		
$\beta_{9.}$	0.947	0.952	0.954	
$\beta_{10.}$	0.947	0.943	0.944	0.937
$\beta_{11.}$	0.946	0.948		
$\beta_{12.}$	0.955		0.929	

Table 1: Coverage proportions for each parameter and covariate combination, showing the proportion of simulations for which the true value of each parameter was contained in its credible interval. Each row corresponds to a parameter and each column corresponds to a covariate. For $i = 1, \ldots, 12$, the vector $\beta_{i.}$ represents model coefficients for cluster i. Blank spaces in the table indicate either a group did not have a certain covariate or a fixed value. For example, the intercept was included in the model for every cluster, so there is no value for π under the column "Intercept."

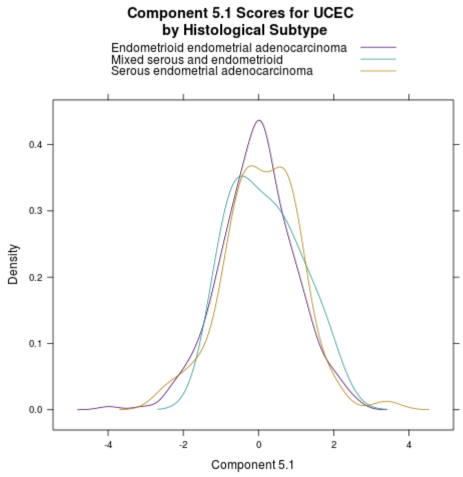

(a)

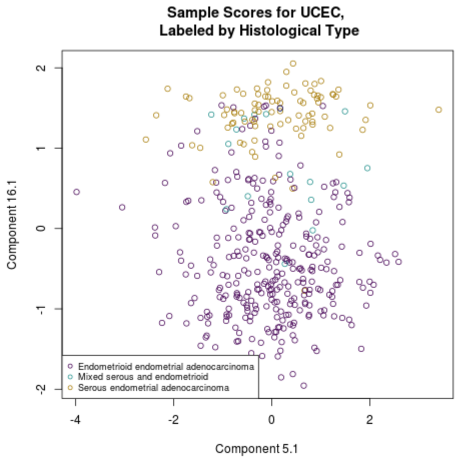

(b)

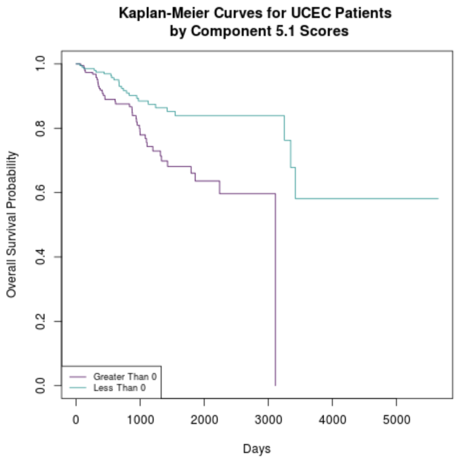

(c)

Figure 1: Figure 1a shows a KDE plot for subjects within each UCEC subtype along component 5.1. Component 5.1 does not appear to explain much variability in these subtypes. A similar deduction can be made from the scatterplot of subjects according to components 16.1 and 5.1 in figure 1b. However, this pattern of variability is marginally associated with survival, as shown in figure 1c, where subjects grouped by their component 5.1 scores appears to have different survival trajectories.

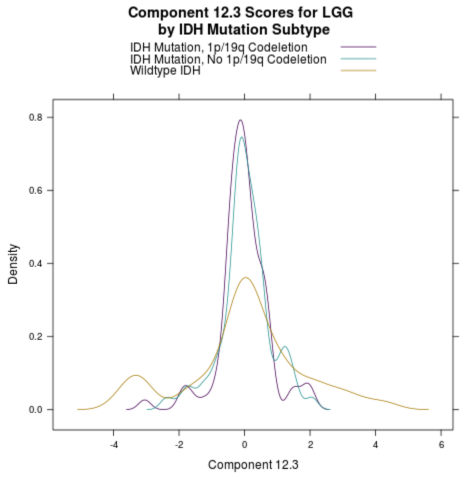

(a)

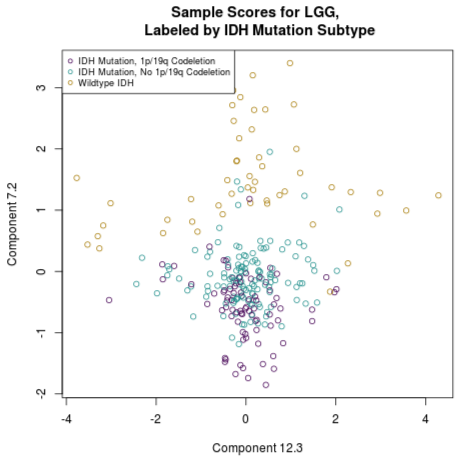

(b)

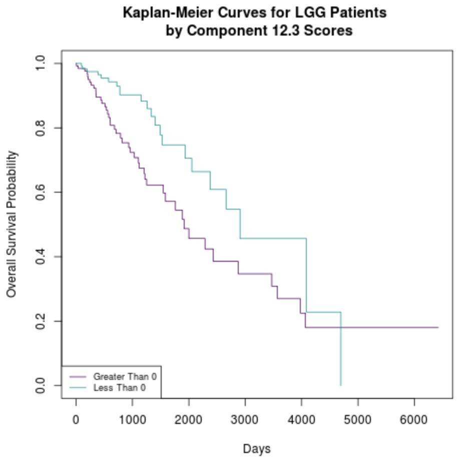

(c)

Figure 2: Figure 2a shows a KDE plot of subjects clustering by their IDH mutation group along component 12.3. This plot shows that this pattern explains some of the variation in IDH mutation status, but as much as component 7.2. Figure 2b shows how subjects cluster along both components 7.2 and 12.3. Despite a less distinct clustering pattern along component 12.3, it appears to have a marginal association with survival, as shown in figure 2c.

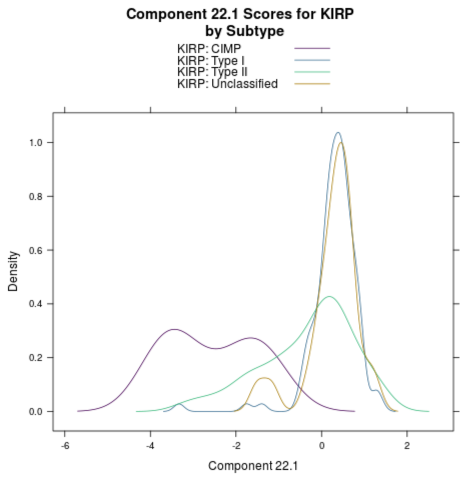

(a)

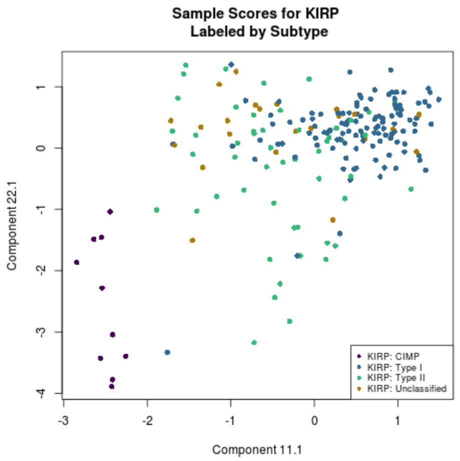

(b)

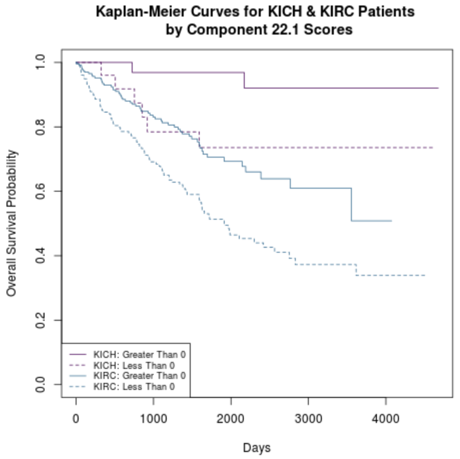

(c)

Figure 3: Figure 3a shows a KDE plot for KIRP subjects along component 22.1 clustering according to their clinical subtype. This demonstrates the CIMP subgroup clusters distinctly from the remaining groups, though not dramatically. Figure 3b shows how KIRP subjects cluster along components 11.1 and 22.1. Both patterns of variability appear to explain variation in the CIMP methylator phenotype in KIRP subjects to differing degrees. Despite an inability to connect this component of variation to any known clinical feature in KICH and KIRC, figure 3c shows that this component is marginally related to survival.

Group	Covariate $j = 1$	Covariate $j = 2$	Covariate $j = 3$
1	0.856		0.826
2	0.919	0.892	0.891
3		0.885	
4	0.926	0.907	
5			0.918
6	0.912		0.924
7		0.904	
8	0.912		
9	0.929	0.899	
10	0.868	0.889	0.875
11	0.926		
12		0.833	

Table 2: Selection accuracy results for the coefficient parameters of each group. Each cell is the proportion of iterations that a particular covariate was correctly included in the model. Each row corresponds to a parameter and each column corresponds to a covariate. For $i = 1, \ldots, 12$, the vector $\beta_{i.}$ represents model coefficients for cluster i. Blank spaces in the table indicate a covariate was not available. For example, group 12 did not have covariates 1 and 3.
